# Facilitated telemedicine for hepatitis C virus: Addressing challenges for improving health and life for people with opioid use disorder

**DOI:** 10.1111/hex.13854

**Published:** 2023-08-28

**Authors:** Andrew H. Talal, Urmo Jaanimägi, Arpan Dharia, Suzanne S. Dickerson

**Affiliations:** ^1^ Department of Medicine Jacobs School of Medicine, Division of Gastroenterology, Hepatology, and Nutrition, University at Buffalo Buffalo New York USA; ^2^ School of Nursing, Division of Biobehavioral Health and Clinical Sciences, University at Buffalo Buffalo New York USA

**Keywords:** hepatitis C virus, stigma, substance use, telemedicine

## Abstract

**Background:**

People who use drugs (PWUD) frequently delay or avoid obtaining medical care in traditional healthcare settings. Through a randomized controlled trial, we investigated facilitated telemedicine for hepatitis C virus (HCV) integrated into opioid treatment programmes. We sought to understand the experiences and meanings of facilitated telemedicine and an HCV cure among PWUD.

**Methods:**

We utilized purposive sampling to interview 25 participants, 6–40 months after achieving an HCV cure. We interpreted and explicated common meanings of participants' experiences of an HCV cure obtained through facilitated telemedicine.

**Results:**

Participants embraced facilitated telemedicine integrated into opioid treatment programmes as patient‐centred care delivered in ‘safe spaces’ (Theme 1). Participants elucidated their experiences of substance use and HCV while committing to treatment for both entities. Facilitated telemedicine integrated into opioid treatment programmes enabled participants to avoid stigma encountered in conventional healthcare settings (Theme 2). Participants conveyed facing negative perceptions of HCV and substance use disorder. Improved self‐awareness, acquired through HCV and substance use treatment, enabled participants to develop strategies to address shame and stigma (Theme 3). An HCV cure, considered by PWUD as a victory over a lethal infectious disease, promotes self‐confidence, enabling participants to improve their health and lives (Theme 4).

**Conclusions:**

Integrating facilitated telemedicine into opioid treatment programmes addresses several healthcare barriers for PWUD. Similarly, obtaining an HCV cure increases their self‐confidence, permissive to positive lifestyle changes and mitigating the negative consequences of substance use.

**Patient and Public Contribution:**

In this study of patient involvement, we interviewed patient‐participants to understand the meaning of an HCV cure through facilitated telemedicine. Participants from a facilitated telemedicine pilot study provided essential input on the design and outcomes of a randomized controlled trial. Pilot study participants endorsed facilitated telemedicine in a testimonial video. They attended site initiation meetings to guide trial implementation. A Patient Advisory Committee (PAC) ensured that patient participants were active members of the research team. The PAC represented patients' voices through feedback on study procedures. A Sustainability Committee supported public involvement in the research process, including educational opportunities, feedback on implementation, and future sustainability considerations.

## 1 INTRODUCTION

People with opioid use disorder frequently experience related infectious diseases, such as hepatitis C virus (HCV) infection. Because of the opioid epidemic, HCV disease burden is the highest amongst the population that uses injection drugs.^1^ Simultaneously, HCV treatment has been revolutionized through the development of direct‐acting antivirals that are highly efficacious with minimal adverse effects.^2^ HCV treatment effectiveness offers an opportunity to eliminate HCV both within the United States and globally.^3^, ^4^ Despite their availability, however, HCV prevalence and incidence continue to increase, and HCV treatment access and uptake remain low, especially among people who use drugs (PWUD). Current literature identifies HCV treatment obstacles for PWUD as poorly controlled substance use, unstable psychiatric conditions, competing priorities, poor relations with healthcare providers, and fear of stigma.^5^

Previous literature has described people with opioid use disorder considering opioid treatment programmes (OTPs) as comfortable and familiar environments with reduced stigma than is typically encountered in general society.^6^, ^7^ Therefore, management of comorbid conditions, such as HCV, integrated into OTPs may improve treatment outcomes. A recent systematic review found that eHealth technologies, which include a few telemedicine studies, show promise largely for HCV screening and diagnosis.^8^ Telemedicine, because of its ability to overcome geographic restrictions on healthcare access, holds promise to expand healthcare delivery to underserved populations. The acceptability and best practices for healthcare delivery through telemedicine to underserved populations, however, remain poorly defined. To overcome telemedicine challenges to underserved populations, such as limited broadband access and unfamiliarity with operating telemedicine equipment, a facilitated telemedicine model was developed. In that model, the entire telemedicine encounter occurs in the safe environment of an OTP. In New York State, methadone dispensing requires frequent in‐person attendance, and the OTP is considered a safe space by PWUD. Telemedicine encounters are facilitated by a case manager who operates the telemedicine equipment and serves as a familiar face to participants. These activities promote trust and confidence in the facilitated telemedicine system. In prior work, the facilitated telemedicine model achieved 93% HCV cure and was well‐accepted by PWUD.^9^, ^10^

We sought to understand the meaning of facilitated telemedicine for HCV integrated into OTPs from individuals who achieved an HCV cure. To gain insight into their experiences, we interviewed participants an average of 19 months after they achieved an HCV cure. By understanding the common meanings, shared practices, and practical advice related to the facilitated telemedicine model and an HCV cure, we seek to inform future clinical care and policy decisions related to HCV elimination.

## 2 METHODS

### 2.1 Setting and design

We had an opportunity to interview study participants from a recently completed randomized controlled trial to assess HCV treatment through facilitated telemedicine (intervention) integrated into 12 OTPs throughout New York State compared to usual care, offsite referral to a liver specialist (control condition).^11^, ^12^ OTPs dispense evidence‐based medication for opioid use disorder, methadone and buprenorphine, in combination with behavioural therapy. The randomized trial utilized the stepped wedge design in which all sites initially conducted the usual care (i.e., referral) arm. After 9 months, a group of four randomly selected OTPs transferred to the facilitated telemedicine (intervention) arm, while the remaining eight sites continued with the usual care arm. After a subsequent 9‐month period, a second group of four OTPs transferred to the facilitated telemedicine (intervention) arm. Finally, after an additional 9 months, the final group of four OTPs transferred to facilitated telemedicine (intervention). During each of the 9‐month periods, participants in the randomized trial initiated HCV treatment, which can last up to 3 months. They were then followed for an additional 3 months to assess for an HCV cure.

### 2.2 Patient and public contribution

This investigation is a study of patient involvement in which patient‐ participants were interviewed to understand the meaning of an HCV cure. Before the randomized controlled trial, we conducted a pilot study to evaluate the acceptability and feasibility of facilitated telemedicine integrated into OTPs for HCV treatment. When the pilot study concluded, we held a focus group and produced a testimonial video of patients' endorsement of facilitated telemedicine. We presented the testimonial video at site initiation meetings for the randomized controlled trial. The testimonial video, along with in‐person appearances from pilot study participants, promoted site recruitment during the randomized controlled trial. To fully represent patients' voices, we established a Patient Advisory Committee (PAC). As active research participants, the PAC identified areas that required clarification and enabled course‐correction of study procedures.^13^ For example, the PAC expressed a concern that participants may not be familiar with telemedicine. To address these concerns, we produced an introductory video to explain the entire facilitated telemedicine process. Other PAC topics of interest included maintaining confidentiality during medication dispensing, compensating patients for completing study activities, and providing hepatitis fact sheets. We honoured the PAC's feedback by actualizing their recommendations. Overall, the PAC was involved in the design and implementation of the randomized controlled trial, as well as improving recruitment, enrolment, and retention.^14^

The PAC also identified the importance of HCV education, a task that was addressed through public involvement with the Chronic Liver Disease Foundation and the American Liver Foundation, two organizations whose primary mission is patient and provider education. Additionally, we involved the Office of Addiction Services and Supports (OASAS), the state agency that governs a network of OTPs in New York State, as well as the corresponding federal agency, the Substance Abuse Mental Health Services Administration, in addressing educational concerns related to HCV. In terms of the trial, OASAS assisted in site recruitment, provided input on the study protocol, and approved study procedures before initiation. We also established a Sustainability Committee, comprised of stakeholders from academic institutions, government agencies, nonprofit organizations, and pharmaceutical and diagnostic corporations. The Committee provided feedback on study procedures and made recommendations for the future sustainability of facilitated telemedicine. The public engagement occurred through educational endeavours conducted in collaboration with the New York State Department of Health, such as the development of a statewide telemedicine workgroup and a telemedicine implementation toolkit (https://ceitraining.org/documents/HCV_Telehealth%20Toolkit_FINAL2023.pdf). These educational initiatives have promoted public engagement of facilitated telemedicine throughout New York State and beyond.

### 2.3 Participant recruitment

We used purposive sampling, consistent with hermeneutic phenomenology study design.^15^ We interviewed 25 participants cured of HCV to understand their experiences of HCV treatment through facilitated telemedicine integrated into OTPs. Study staff or members of the PAC referred potential interviewees. One of two study staff members interviewed participants individually after obtaining written informed consent. We interviewed participants 6–40 months (average 19 months) after obtaining an HCV cure as we sought to interview participants from all 11 study sites participating in the interview substudy. The institutional review boards at participating institutions approved the study.

### 2.4 Interview conduct

During the interviews, we inquired about participants' experiences of HCV treatment through facilitated telemedicine in OTPs. We used open‐ended questions and interview prompts to facilitate discussion and elaboration. We asked participants to cite specific examples of the care they received, including what was helpful and not helpful in their pursuit of HCV treatment. We also asked them to make suggestions for future deployment of the facilitated telemedicine model. The interviews were recorded and transcribed using Zoom. Study staff deidentified and verified all transcriptions by comparing their accuracy against the recordings. Interviewers confirmed the final transcript version before analysis. We analyzed the confirmed, deidentified text version.

### 2.5 Qualitative analysis

We used hermeneutic phenomenology to understand human situations as they were experienced within a context of time, place, and situational influences, such as obtaining an HCV cure through telemedicine integrated into an OTP.^15^, ^16^, ^17^ We utilized hermeneutic phenomenology to formulate interview questions, interpret interview text, and explicate common meanings to generate in‐depth understanding of participant experiences of facilitated telemedicine for HCV management to inform public health policy. During data collection, the researcher questions and cocreates with the participant an opening for new thinking and possibilities. Thus, the researcher attempts to situate themselves in the experience of the participant to understand its meaning from the participant's point of view. The researcher must acknowledge that background practices are revealed through the language of experience. Thus, the interviews provide an intelligible meaning to understand the situatedness of the healthcare system for patients with opioid use disorder and HCV infection. We chose hermeneutic phenomenology, which seeks temporal meaning and situational context, over grounded theory, which focuses on theory development through understanding of basic social processes. Similarly, we chose the approach over psychology‐informed interpretative phenomenological analysis, which emphasizes constructs of cognition.^15^, ^18^, ^19^, ^20^, ^21^

**Figure 1:**
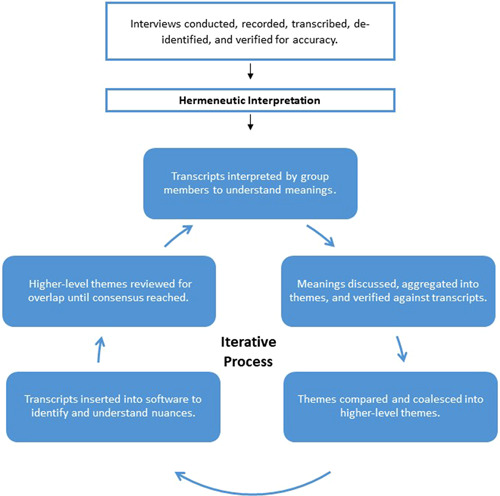
Interpretation of transcripts of participants experiencing facilitated telemedicine for hepatitis C virus treatment. The figure illustrates the hermeneutic phenomenological approach utilized to understand and code the meanings of hepatitis C virus treatment integrated into an opioid treatment programme.

Interpretation of themes was revealed through experiences, as captured through the participant interviews. All individual interview transcripts served as the data for interpretation. The analysis team included the study's principal investigator (i.e., a physician‐scientist liver diseases expert), a professor of nursing who is an expert in hermeneutic qualitative analysis, and the project manager. The hermeneutics expert guided the analysis. The team interpreted the texts in a reflective process that followed iterative steps^15^, ^22^ (Figure 1). Ultimately, all team members agreed on the interpretation, related themes, and constitutive pattern as a warranted, coherent, and comprehensive interpretation of participants' experiences of HCV care. We expressed rigour in accordance with de Witt and Ploeg's^23^ framework of balanced integration, openness, concreteness, resonance, and actualization. We balanced integration by bringing together the participants' voices and the researchers' interpretations. Study results, as explicated in themes and a constitutive pattern, use both verbatim excerpts and interpretive explanations. Openness occurred through a systematic process of auditing the interpretive decisions, returning to the text for verification and consensus in the group analysis as recorded in reflexive journaling. Concreteness in the findings was expressed by providing context to situate the audience in the phenomenon by using examples that resonate with life experiences. Resonance was expressed when the audience had an intuitive grasp of the research concept through reading verbatim examples that support the findings. Actualization occurs in future readings of the findings that achieve resonance for the readers.

## 3 RESULTS

Our interpretation includes four themes and one constitutive pattern to explicate ‘Facilitated Telemedicine for HCV: Addressing Challenges for Improving Health and Life for People with Opioid Use Disorder’. Themes illustrate patients' experiences of (1) embracing facilitated telemedicine in the OTP; (2) committing to the OTP and HCV treatment; (3) facing negative perceptions of HCV and substance use disorder; and (4) engaging in HCV treatment to improve health.

### 3.1 Theme 1: Embracing facilitated telemedicine in the OTP

Participants viewed their OTPs as supportive and trusting environments, qualities that extended to the telemedicine encounter and provider. Overall, participants were enthusiastic about healthcare delivery through facilitated telemedicine. One participant explained that onsite facilitated telemedicine for healthcare delivery mitigated the apprehension he/she faced as a substance user when referred to offsite healthcare settings. ‘I would be more comfortable at my methadone clinic than going to a primary care doctor or the emergency room’. Participants elaborated how facilitated telemedicine expanded healthcare access in a convenient setting and removed barriers to care, such as transportation:

I honestly wouldn't have done it [HCV treatment]; I would have put it off. Telemedicine saves so much time, as opposed to getting in the car, going to the doctor, waiting in line, [and] checking in. It's been a wonderful experience.

Participants were initially anxious about telemedicine encounters. After the study‐supported case manager provided HCV education that also addressed misconceptions and assured privacy and confidentiality during the encounter, they became more accepting of facilitated telemedicine. One participant elaborated:

They didn't push anything, they explained how it works and broke it down in [an] understandable way. They're very discreet… It's a one‐on‐one type of atmosphere.

The case manager was also an onsite telemedicine facilitator as well as a convenient go‐to person who alleviated study participants' fears and concerns, encouraging and integrating patient‐centred care into the OTP for hepatitis C*.*

When I got my [test] results that I have hepatitis C again, [the case manager] called me to the office and spoke to me in person [about] treatment. She explained [that the] new medicine is not the old medicine.

The case managers leveraged the caring atmospheres of the OTP for effective facilitated telemedicine:

The case manager made me feel the most comfortable that I've ever felt, even though they knew I was an IV drug user. They actually will take your hand if you're having a bad day and give you a hug. Little things like that, they're constantly smiling, telling you good things about how you're doing, showing you the levels [of the virus], that just makes you feel good.

Participants also valued the integration of HCV treatment within the OTP that provided seamless interactions between the case manager and OTP staff:

Everybody that participated, doctors, workers, I couldn't ask for a better group. Always checking on you… I still tell people to this day how excellent it was.

Participants also valued onsite care and summarized advantages that are related to colocation of HCV care within the OTP:

Even if you're somebody that goes to the clinic every day, being able to get your [HCV] dose at the window is perfect, because a lot of people forget about taking their dose. You're already there and you're already taking your [substance use disorder] medication.

### 3.2 Theme 2: Committing to the OTP and HCV treatment

Participants described their experiences of substance use as losing control of their lives. One participant described his substance use as a primary need ‘to feel a high. [You] will spend all your money, [and] the next day, you're broke’. When experiencing substance use disorder, participants also struggle with symptoms of anxiety and depression that manifest in their attitudes of impatience, short attention spans, and ‘loud and vulgar’ behaviour. Once participants decided to pursue treatment for substance use disorder, they enroled in the OTP ‘trying to get clean and sober’, as the programme provides methadone as an evidence‐based treatment to manage withdrawal symptoms and counselling to address mental health issues, as one participant explained:

There's a lot of ways to beat addiction. It is not just ‘give me a bottle of methadone and let me be on my way’. Sometimes people need to talk… Groups are very supportive.

One participant explained the need to commit themselves to treatment for both substance use and HCV, ‘It's not mandatory, most of the people [are] there on their own will. Help is there, but [it is] up to you’. Participants depicted the wide array of OTP services in addition to methadone and counselling.

[They] can refer you to mental health services… They also have job training programmes… Having a counselor to help with [individual] needs… one‐on‐one is the therapeutic value.

Almost uniformly, participants considered the OTP a destigmatizing community where they felt positive reinforcement from frequent contact with OTP staff. Participants appreciated that OTP staff, including counsellors who address them by their first names, nurture mutual respect and understand the nature of substance use disorder:

Everybody there really cares and it's personal, we're not just numbers. I can trust them enough to speak [about] what I was going through, and they helped me out a lot.

Counsellors provided the first line of support, not only for substance use treatment, but they also facilitated patient engagement in HCV treatment through facilitated telemedicine. The net effect of the OTP is to promote recovery from substance use disorder and co‐occurring conditions, such as HCV, and improve self‐awareness, ‘[The treatment goal] is less of the addiction part and more of *how to move on* from it’. One participant shared the change in his/her life brought about by opioid use disorder treatment, ‘When I was using [drugs], I would try to cut corners. Not using [drugs] brings stability to other aspects of my life’. The process of committing to substance use and HCV treatment in the OTP and moving to healing can be long and arduous as one participant reflected:

It is hard to get someone to come to the programme but stay in the programme as long as it takes because the OTP is an intricate part of your life.

### 3.3 Theme 3: Facing negative perceptions of HCV and substance use disorder

Participants referred to themselves as ‘addicts’, a learned label and attitude associated with substance use that reinforces negative self‐perceptions. This attitude was revealed in the language that participants used to refer to themselves as ‘dirty’ or ‘clean’ depending on their current state of substance use, as one participant reflected:

A lot of addicts feel the same way [about their substance use]… You can't imagine the horror that I go through… It's hard to explain unless you're familiar with addiction personally.

#### 3.3.1 Subtheme 3a: Confronting poor relations with external healthcare providers

Participants revealed that one of the advantages of facilitated telemedicine situated in the OTP is that it permits them to circumvent the obstacles they face in traditional healthcare settings where they are ‘labelled as a drug user’. One participant related a similar sentiment when seeking healthcare in a conventional healthcare setting*:*

When they found out that I was an IV drug user, the whole atmosphere changes… And that's one of the reasons I never sought treatment for [HCV].

Another participant cited a story about seeking care for injury‐associated pain in the hospital where they perceived hospital workers' prejudice when checking the opioid registry.

[I was] red flagged every single time. When you try to get pain pills, [they think] you fake it because you can't afford the drugs on the streets. You have to go by the [healthcare] system [rules].

Another participant shared his/her experience with a provider that demonstrated a misunderstanding of methadone's role to alleviate withdrawal symptoms:

[The dentist] actually refused to give me Novocain because I was on methadone… Obviously I switched dentists… A lot of it is knowledge‐related and [lack of] education.

The shame and stigma of substance use led participants to delay or entirely avoid seeking healthcare in conventional settings. As the participants learned of the negative consequences of disclosing their methadone status to providers, they also realized that practicing nondisclosure was a strategy to avoid the shunning when seeking healthcare in conventional settings:

I don't usually bring it up, unless I absolutely have to… If I'm in a medical setting, obviously I want them to know what medications I'm on, but in my normal discourse with people, I don't bring up that I'm on methadone.

#### 3.3.2 Subtheme 3b: Enduring negative perceptions of substance use disorder in society

Participants related experiences in society where they were judged for their substance use disorder as a moral failing, a misunderstanding of the basis of substance use disorder as a physiologic medical condition, as one participant summarized:

A lot of people have no education about drugs, all they know is from movies… Drug [addiction] is a disease… People think it's not a disease or that this is what you want to do, and you can stop at any moment with no problem. And if you don't want to stop, then you are a drug addict, you're a bad person and selfish. Of course, it's wrong.

A participant shared his/her view of society's assumptions about substance use as a psychological condition that persists even if someone is well on their way to recovery. ‘It is in their mind… They assume that what you were is still a part of you now’. Another participant concurred and clarified society's misunderstandings of people with substance use disorder:

[Others have] misconceptions about drug abuse, they think an addict is somebody who hangs [out] in the street all day. I work, I've worked all my life. I come from a family that worked, I went to college. Yet it's the stigma, the attitudes and preconceptions that come with drug addiction.

One participant also elaborated on societal misconceptions surrounding the treatment of substance use disorder, specifically methadone:

[Methadone] does raise eyebrows… I'll have to explain about being on [methadone], it's not smoking crystal meth… Sometimes, that's what police officers will think… You have to actually say ‘I am *in* a methadone clinic’.

Indeed, participants learned to state that they were ‘***in a methadone programme***’ (i.e., indicating adherence to an evidence‐based treatment) as opposed to being ‘***on methadone***’, which is an effective communication strategy with law enforcement by emphasizing that methadone is an evidence‐based treatment for a physiological condition.

Thus, although opioid use disorder affects every aspect of participants' lives, through treatment of substance use disorder and co‐occurring conditions, such as HCV, participants develop self‐awareness of the negative attitudes of those around them and begin to develop strategies to mitigate the shame.

#### 3.3.3 Subtheme 3c: HCV diagnosis as shameful

As participants progress through treatment for opioid addiction, they develop self‐awareness of the misunderstandings of substance use disorder, associated comorbid conditions such as HCV, and of the negative attitudes held by people outside of the OTP. ‘They think that because you're on methadone, you're sh**, you're dirt, you're lower than low’.

Participants' narratives portrayed feelings of embarrassment and doom related to a diagnosis of HCV. ‘[HCV] is not only dirty but disgusting, because people may think that anyone that got [it] was using needles for drugs’. Because of these sentiments, some participants initially delayed seeking HCV treatment. ‘I didn't tell anybody, no family members or anything’. Because of HCV‐related shame and stigma, patients do not feel comfortable discussing an HCV diagnosis with others and voiced their concerns related to privacy and confidentiality surrounding an HCV diagnosis and treatment.

### 3.4 Theme 4: Engaging in HCV treatment to improve health

Several participants were motivated to pursue HCV treatment because they viewed HCV infection as a lethal disease, and they personally knew friends and family who died of HCV:

I don't want to pass hepatitis C to nobody… I know that hepatitis C is hard. I got family, my niece passed away [from] hepatitis C… I [want] to get the treatment for hepatitis C.

Participants recounted stories of prior HCV treatment regimens (i.e., interferon) that were arduous and highly ineffective. The education that was provided during the study informed them of modern HCV treatment regimens with high efficacy and minimal side effects, potentiating an HCV cure and hope for the future:

Before I knew they had a cure for HCV, I thought I was doomed… If I still got hepatitis C, I am not going to last long. It makes no sense to see a doctor because the doctor is not going to do anything for me. Until you guys [study personnel] showed up… I was tired, I was fatigued. It was a lot of symptoms going on with me.

Furthermore, participants commented on how rapidly HCV treatment, once initiated, improved their symptoms and quality of life:

[It helped] tremendously, within a month and a half, I started seeing results. I started feeling different… My legs are moving faster… I feel amazing.

Achieving an HCV cure enabled participants to start a new beginning in their lives by ‘planning new goals with a brighter outlook’. The HCV cure also enabled participants to improve their self‐confidence, ‘It's definitely made me feel a lot better, more confident’. Increased energy and newfound self‐confidence enabled people who achieved a cure to pursue many new directions. As one participant commented:

HCV treatment was a catalyst for getting life under control and for changing many things in my life. I'm just glad that they were able to find a cure for it.

Several participants explained specifically how the cure affected the rest of their lives. For example, one participant appreciated the cure as it decreased anxiety:

I don't have the thought in my head anymore that I got this thing in my body that's killing [me]… I think it is a godsend. In eight weeks, you could help somebody prolong their life.

Another participant shared his/her newfound joy in life after having been cured of HCV:

I wake up every morning happy, joyful, [drug‐]free. I wake up not worrying about the pain, the suffering, the shakes, the headaches, the sweats, the disillusions, the lies that go through my mind of getting one more [high]. This programme granted that. Freedom in choices for me today!

Several participants discussed the importance of supporting and providing others the chance to use the facilitated telemedicine model. Participants discussed sharing their successes with others via ‘a peer pipeline [and] spreading the word about HCV treatment to help others, anything to help mankind’. The peer pipeline was emphasized as a way to inform others of the importance of an HCV cure:

I think [by getting] the word out, a lot more people would do the treatment… I have five people who have reached out to me that want to get treatment.

Participants vocalized their understanding of the need to speak freely about HCV treatment, ‘Telling others about treatment; awareness of having knowledge [that can] be disseminated to benefit others’.

Besides the peer pipeline, participants recommended not only expansion of facilitated telemedicine to other methadone programmes, but to other substance use treatment programmes as well.

A lot of people with this problem are not on methadone, they're not aware of it, or their methadone clinic doesn't deal with it. There [should be] a way [for] methadone [or] addiction systems [to share] the fact ‘Hey, there is something you could do’.

Not only did participants recount their own experiences of attempting to convince family members, peers, and their programmes of HCV treatment benefits, some participants viewed an HCV cure as a steppingstone toward a career change. One participant commented, ‘I'm trying to become a counsellor because I feel I have so much to offer’.

### 3.5 Constitutive pattern

#### 3.5.1 Facilitated telemedicine for HCV: Addressing challenges for improving health and life for people with opioid use disorder

**Figure 2:**
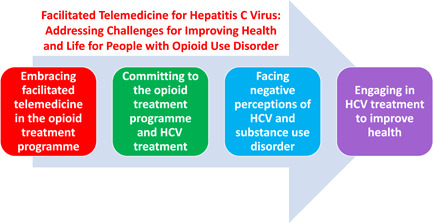
Study themes as revealed by participant interviews. Integrating facilitated telemedicine for hepatitis C virus treatment into opioid treatment programmes facilitates commitment to substance use and HCV treatment enabling participants to mitigate negative perceptions of these conditions and leading to improved health.

Participants underscore the convenience and confidentiality of facilitated telemedicine integrated into the OTP, providing access to patient‐centred care, and circumventing external providers. They embrace facilitated telemedicine as promoting a therapeutic relationship between patients and telemedicine providers. These relationships are facilitated by a case manager who builds trust with patients, answers their questions, and facilitates the telemedicine encounter (*Theme 1: Embracing facilitated telemedicine in the opioid treatment programme*). Committing to treatment for substance use disorder and HCV in the OTP is seen as an initial step in the recovery process. Substance use disorder treatment stabilizes participants' lives and enables them to partake of advantages offered by the OTP, such as mental health treatment, job training, and facilitated telemedicine for HCV (*Theme 2: Committing to the opioid treatment programme and HCV treatment*). Substance use disorder and its associated shame are pervasive in all aspects of participants' lives, including from providers external to the OTP, from society, and from an HCV diagnosis. Focusing on the physiological basis of substance use disorder and the evidence‐based therapy provided in OTPs can promote self‐awareness and the development of strategies to address stigma and shame (*Theme 3: Facing negative perceptions of HCV and substance use disorder*). Participants describe feeling increased energy after achieving an HCV cure, encouraging positive lifestyle changes, and securing a victory over a life‐threatening infection. Participants convey that the cure promotes self‐confidence, inspiring activities that improve their own lives (*Theme 4: Engaging in HCV treatment to improve health*). These insights afford an important understanding of the significant contribution of an HCV cure to improving health and life for people with opioid use disorders. Additional supportive quotations are listed (Table 1) (Figure 2).

**Table 1:** Additional supportive participant quotations.

Themes	Meanings	Examples
Embracing facilitated telemedicine in the opioid treatment programme	Convenience	‘I think it [telemedicine] was great for me because I don't like doctors, I don't like hospitals, and to be able get this done while coming into the clinic was wonderful’. ‘Telemedicine is a good experience, just like [the doctor] was there, easy conversation’. ‘I know it's going to take a while for telemedicine to [go] into effect, but it would be wonderful because so many more people will get treatment’.
	Case manager integrating into the opioid treatment programme	‘Phlebotomist and case manager were very helpful. They were very knowledgeable; they were gentle with me because I am an emotional type of person’. ‘[We] talked about it, and I was scared. He [case manager] told me I was going to be all right. He helped me get through it. He kept on telling me “You're going to be fine”’. ‘I don't have to go [to outside providers] and wait two hours just to [be] sent home with ibuprofen and a cup of water. I could call the [case manager] to explain what was going on, and I really appreciate that’.
	Valuing care integration and the team	‘I felt that they were involved with me every step of the process… When I had to give blood, they told me exactly what it was for. They told me how I should be feeling at this point, and three weeks down the road, how I should be feeling’. ‘The first moment, I [was] scared. After I started treatment, I felt good. I feel comfortable with the medicine. The doctor explained everything, and I understood I needed the treatment, and I said “let's go”’. ‘Confidentiality, as far as people not knowing my situation, they're very discreet… They do a really good job not discussing other clients' situations. It's a one‐on‐one type of atmosphere’. ‘The counselors would be walking around [and] if they see you, they would say, “I just wanted to say hi” and “When you are done getting dosed, can we sit down for two minutes? I just want to know how your day is going”. They are involved 100%’.
Committing to the opioid treatment programme and HCV treatment	Gaining trust	‘I trust the [OTP] community, but it took some time for me to trust them. [At first] I didn't want to hear nothing… I just wanted to come in and leave. The counselors took their time; they were very patient, even when I was disrespectful… They changed my life around, the counselors, because I was heading to a graveyard’. ‘The methadone clinic is the best place that I've ever been to. This is the most comfortable I've ever been in a setting with so many people’.
	Substance use disorder treatment	‘Once I got addicted to the pain pills… it took years of counselling and medication… If I were to stop taking methadone [which] I am nervous to do… I feel that I would go right back to the opiates’. ‘I wish you had known me 20 years ago. You would be amazed at the transformation, the change through this programme. When I first came here, I was a total mess’. ‘I had a negative attitude about myself, people around me, doctors and nurses. I took a good look at myself, and I went back to college, and I graduated. It was a counsellor in the methadone programme that changed me. I never looked back. I changed my ways, and it was for the better. And this [programme] made me the person I am today’. ‘The experience with the methadone is absolutely phenomenal compared to what I was doing on the street’. ‘It's a great programme and saved my life, methadone is turning my life around. I've been here two years, hard to come, harder to stay, [stay] as long as it takes’.
Facing negative perceptions of HCV and substance use disorder	Confronting poor relations with external healthcare providers	‘Going to the hospital and knowing you're on methadone, they think you're a heroin addict. People try to hide it but they look at you a little different, “Oh, here comes another addict”’. ‘If it's somebody new [physician] and with addicts, I get the feeling that doctors think that we're always doctor‐shopping or trying to get drugs… Sometimes it happens, and sometimes it's uncomfortable’. ‘I've been with my doctor a couple years now, but when they find out you're on methadone, they don't want to give you the meds that you need’. ‘When I go to the emergency room, I need to tell them [about methadone]. They need to know that I'm on methadone because it could be a contraindication to another medication’. ‘I've spoken to other doctors who tell me that addiction providers are considered the bottom of the totem pole [in] the medical field… How much training does the doctor have on drug treatment? Misconception about drug abuse, period’.
	Enduring negative perceptions of substance use disorder in society	‘I have had an incident when I said, “I'm on methadone” and they [police] look you up and down “what the hell kind of drug are you talking about? Are you smoking crystal meth?” and I've been pulled out of my vehicle… It makes me slow down and think’. ‘Certain people, I won't talk to them, and they don't talk to me. People get off the train, and we're having coffee on the corner, some of them look at us and think that we're all addicts, we're not’. ‘I say, "I'm in the methadone programme"… They know exactly what you're talking about… If you [say] I'm on methadone, they start questioning you, and they don't know what you're talking about… I'm telling people that I am in the programme, and I'm physically in here, not on it’.
	HCV diagnosis as shameful	‘I was never an IV drug user, so how did I get hepatitis? The doctor told me you could get it by sharing a straw. I automatically assumed that [HCV] went with IV drug use’. ‘Nobody told me there was a treatment and that I can be cured. I thought it was something I had to live with for the rest of my life’.
Engaging in HCV treatment to improve health	HCV treatment motivation	‘Now, when you're tired, you don't think anything's wrong. But once you find out you have it [HCV], they can get you on the right medication, get you out on the right path, and you will be fine’. ‘I have kids and I didn't want to have an old razor laying around… I wanted to get my health back on track, so I said yes to the study [and HCV treatment]’. ‘Overall, it was a great experience. I had no problems with the medication. I was very fearful going into it because of the past, how the treatment used to be literally like chemo. I don't think people understand how different this new treatment is’. ‘Lots of patients [are] still suffering from hepatitis C, and they [are] not aware of the new medicine… [they] still think that it's the old, long, heavy treatment’.
	Benefits of an HCV cure to the individual	‘I got more energy. I feel rejuvenated… I feel 100% better… By getting cured from hepatitis C that prolonged my life’. ‘The nurse said “you no longer come up as infected with hep C” so that was a great feeling, like a bond to not expose myself again. I know people that have… I told them, listen, it works, and you'll be free of that and then live a healthier life’. ‘Before the medicine, I was tired all the time. I didn't want to get out of bed to do the things that I love: fishing, hunting, camping, or going out to dinner. Now, with methadone [and] hepatitis treatment being over, I feel amazing. I'm writing a book on my life… I'm 483 pages into it, and I just need to get a publisher. I'm getting married next month; I'm buying a house to fix up for me and my wife, and things are going the way that they should be’. ‘Right now, my fiancé's making scrambled eggs, steak, potatoes, and French toast. We have our goals, we have our days planned out. Before I would be lying in bed, and she would have to bring breakfast to me because of the hepatitis and how much it takes out of you. Everything's back on track, and I feel amazing’.
	Benefits of the cure to others	‘I wanted to help research. It affects [people's] private lives, especially if you have a family and you don't them want your husband or wife to get sick’.

Abbreviations: HCV; hepatitis C virus, OTP; opioid treatment programme.

## 4 DISCUSSION

Participants revealed how facilitated telemedicine expanded healthcare access in the OTP, which they consider a ‘safe space’ that is supportive, trusting, and destigmatizing. Similarly, other research described OTPs as accepting and comfortable environments.^24^, ^25^ Trust between study participants and OTP staff largely circumvents the stigma encountered in traditional healthcare settings, similar to findings reported previously.^26^ Case managers built upon the existent trust to become integrated into the OTP to form relationships with participants that not only facilitated telemedicine encounters but disseminated knowledge and addressed participants' concerns.^27^ These observations are similar to research on trust forming the basis for a therapeutic alliance that includes an affective bond, agreement on goals, and assignment of tasks.^28^, ^29^, ^30^ The patient‐centred approach of facilitated telemedicine is also consistent with a recent American College of Physicians recommendation to offer ‘all patients the ability to receive care, when and where they need it’.^31^

Although substance use disorder is a physiologic medical condition, participants face perceptions of shame due to negative assumptions about their substance use. They perceived HCV infection as ‘dirty’, resulting in additional anxiety and shame. Participants described experiences of humiliation and shame that add nuanced understanding to other research describing stigma leading to low self‐esteem, social isolation, and reduced intimacy.^32^, ^33^, ^34^ Our interpretations of participants' experiences concur with others who reported that people with opioid use disorder often interpret society's views of substance use as a moral failing and that providers' external to the OTP perceive people with opioid use disorder as irresponsible and nonadherent to medical care.^35^, ^36^ People with opioid use disorder are often viewed as dangerous and unpredictable; consequently, they are treated with humiliation and shame.^37^, ^38^ Participants' stories indicated that accepting substance use disorder as a medical condition and committing to the OTP are initial steps toward coping with the disease. The negative connotation of an HCV diagnosis also can limit people with opioid use disorder engagement in HCV care.

The consequences of substance use disorder, including experienced and anticipated shame, interfere with the pursuit of HCV treatment. In the literature, shame or stigma is conceptualized as a process of separation stemming from discrimination against individuals who exemplify characteristics outside the norm.^35^, ^37^, ^39^ Similarly, the philosopher M. Heidegger explains that everyday societal expectations of ‘the “they”’ (i.e., social norms) are taken‐for‐granted expectations (e.g.,) that people with opioid use disorders are unworthy. ‘The “they”’ itself prescribes a way of interpreting the world (p. 167) that limits the possibilities of others.^16^ As exemplified in the participant interviews, substance use disorder shame (i.e., stigma) can originate from self‐perceptions and perceptions of others in healthcare institutions and society. Self‐stigma is the internalization of blame, low self‐worth, and lack of confidence that may diminish self‐esteem and prolong treatment for substance use disorder.^40^ Participants often avoided or delayed medical care to evade perceptions of substance use disorder shame with potential adverse health consequences, including death.^24^, ^33^, ^41^ Participants indicated that as they progress through substance use disorder treatment, they learned how to navigate, and in many cases mitigate, negative attitudes (Table 2).

**Table 2:** Patient experiences of challenges and strategies to overcome challenges for hepatitis C virus treatment pursuit.

Themes	Challenges	Strategies
Embracing facilitated telemedicine in the opioid treatment programme	Distrusting the healthcare system. Lacking knowledge and understanding of telemedicine.	Seeking a family‐like community environment in which to pursue treatment for substance use disorder and HCV. Promoting a therapeutic alliance with providers with regular communication and positive reinforcement. Benefitting from supportive case management throughout the HCV treatment course. Educating and familiarizing patients about telemedicine.
Facing negative perceptions of HCV and substance use disorder	Confronting poor relations with external providers Shaming experiences as a barrier to seeking treatment in traditional healthcare settings (i.e., emergency department, primary care). Disclosing substance use status to healthcare providers engendering negative feedback and shaming.	Using situational awareness and nondisclosure if necessary. Communicating effectively to reflect substance use disorder treatment goals, for example, ‘in a methadone programme’ versus ‘on methadone’. Educating and training providers to understand substance use disorder treatments and the effects of stigma. Supporting HCV care integrated into the OTP. Evaluating consequences of social structures supporting disparities.
	Enduring negative perceptions of substance use disorder in society Experiencing the misconception of methadone as just another drug. Limited public knowledge of substance use disorder as a medical condition, that is, blaming, shaming as moral failures.	Educating others on substance use disorder as a medical disease. Education on MOUD and counselling as effective, evidence‐based treatments. Increasing public awareness of the experiences of shaming due to substance use, including in law enforcement and the criminal justice system. Using appropriate language to describe substance use disorder treatment.
	HCV as shameful Believing misconceptions of substance use disorder and HCV treatments. Recognizing the negative view of HCV. Knowing others who have died from HCV.	Gaining awareness of substance use disorder as a medical disease. Gaining knowledge of innovative treatment modalities for achieving an HCV cure. Maintaining privacy and confidentiality regarding HCV. Changing policies to support HCV treatment as integrated care within the OTP.
Engaging in HCV treatment to improve health	Believing that HCV treatment is unsafe and ineffective (misconceptions).	Listening to successfully treated peers. Improving self‐confidence, self‐worth, and overall wellness. Obtaining an HCV cure is an accomplishment indicative of progress in addressing substance use disorder. An HCV cure promoting improvement in overall health.

Abbreviations: HCV, hepatitis c virus; OTP, opioid treatment programme; MOUD, medication for opioid use disorder.

Participants also described how an HCV cure was considered a victory, promoting self‐confidence, and enabling the pursuit of beneficial activities for themselves and others. Increased self‐awareness and self‐confidence promoted the development of strategies for participants to address health and life issues, consistent with observations by others.^42^ Participant testimonies and stories give voice to narrative messaging, the portrayal of people with opioid use disorder as successfully treated, asymptomatic, and leading healthy lives. Others have shown that narrative messaging can lead to decreased discrimination against people with substance use disorder.^35^, ^43^ Our participants detailed how an HCV cure contributes to improvement in health and life, thereby potentially mitigating, at least in a small way, negative self‐perceptions of HCV and substance use.

Our study has several strengths. One is the specific elaboration of the manifestations of substance use disorder shame (stigma) (i.e., self, HCV, healthcare, and society) and how these were addressed through facilitated telemedicine and an HCV cure. Addressing specific stigma components provides nuanced understanding, addressing the critique of a recent thematic synthesis that identified that previous investigation of stigma in HCV has largely been assumed and implicit.^30^ Second, we interviewed participants 6–40 months after achieving an HCV cure that enabled us to assess its long‐term significance. Third, we utilized hermeneutic phenomenology for the interpretative analysis to understand the meaning of participants' experiences of HCV care through facilitated telemedicine integrated into the OTP. The approach provided insight into participants' meanings of the HCV cure, including mitigating HCV shame as well as improving health and life. We maintained analytical rigour through the recruitment of participants from multiple sites to understand the nuances of facilitated telemedicine. One limitation is that we only conducted interviews on participants who achieved an HCV cure, not untreated individuals.

## 5 CONCLUSIONS

Participants' stories described how the trusted environment of the OTP promotes substance use disorder treatment. Facilitated telemedicine, along with the integrated case manager and interdisciplinary team of OTP staff, provide patient‐centred care for HCV, while simultaneously addressing participants' concerns. The culmination of these activities, an HCV cure, promotes participants' self‐confidence, enabling the pursuit of activities to improve their health and their lives. Practical advice and best practices related to facilitated telemedicine may be informative in expanding future clinical care and policy decisions related to HCV elimination.

## AUTHOR CONTRIBUTIONS

**Andrew H. Talal**: Conceptualization; data curation; funding acquisition; investigation; project administration; resources; supervision; validation; visualization; writing—original draft; writing—review and editing. **Urmo Jaanimägi**: Data curation; formal analysis; writing—original draft; writing—review and editing. **Arpan Dharia**: Data curation; formal analysis; visualization; writing—original draft; writing—review and editing. **Suzanne S. Dickerson**: Conceptualization; data collection; methodology; resources; software; supervision; formal analysis; investigation; visualization; writing—original draft; writing—review and editing.

## CONFLICT OF INTEREST STATEMENT

Andrew H. Talal has received honoraria and funds to his institution from Gilead Sciences and Abbvie Inc. The other authors declare no conflict of interest.

## ETHICS STATEMENT

This study was approved by the University at Buffalo Institutional Review Board (IRB) and by IRBs at each of the sites where the study was conducted. All participants provided written informed consent before study participation.

## Data Availability

The data that support the findings of this study are available from the corresponding author upon reasonable request.
